# Localized Langerhans cell histiocytosis masquerading as Brodie's abscess in a 2-year-old child: a case report

**DOI:** 10.17179/excli2015-713

**Published:** 2016-01-18

**Authors:** Wei-Fang Chang, Yi-Chih Hsu, Yi-Der Wu, Chun-Lang Kuo, Guo-Shu Huang

**Affiliations:** 1 Department of Radiology, Tri-Service General Hospital, National Defense Medical Center, Taipei, Taiwan; 2 Department of Radiology and Nuclear Medicine, Zuoying Branch of Kaohsiung Armed Forces General Hospital, Kaohsiung, Taiwan; 3 Department of Radiology, Cathay General Hospital, Taipei, Taiwan; 4 Department of Orthopedic Surgery, Taoyuan Armed Forces General Hospital, Taoyuan County, Taiwan

**Keywords:** knee pain, Langerhans cell histiocytosis, Brodie's abscess, penumbra sign, differential diagnosis

## Abstract

Langerhans cell histiocytosis (LCH), formerly known as histiocytosis X, refers to a spectrum of diseases characterized by idiopathic proliferation of histiocytes that produce either focal (localized LCH) or systemic manifestations (**Hand**-**Schüller**-**Christian disease and Letterer**-**Siwe disease**). Localized LCH accounts for approximately 60-70 % of all LCH cases. Osseous involvement is the most common manifestation and typically involves the flat bones, along with lesions of the skull, pelvis, and ribs. Localized LCH in bone shows a wide spectrum of clinical manifestations and radiologic features that may mimic those of infections as well as benign and malignant tumors. The diagnostic imaging findings of localized LCH are also diverse and challenging. The penumbra sign is a common and characteristic magnetic resonance imaging (MRI) feature of Brodie's abscess, but is rarely seen in localized LCH. In this report, we describe a case of localized LCH misdiagnosed as Brodie's abscess in a 2-year-old child based on clinical symptoms, laboratory findings, and pre-diagnostic MRI findings (penumbra sign). Therefore, the penumbra sign is not sufficient to clearly establish the diagnosis of Brodie's abscess, and the differential diagnosis of localized LCH should be considered when a child with an osteolytic lesion presents with a penumbra sign.

## Introduction

Localized Langerhans cell histiocytosis (LCH) of the bone is a relatively rare, primary disease of childhood characterized by clonal proliferation of histiocytic cells and variable biologic behavior (Hashmi et al., 2012[7]). The skull is the most frequently involved bone in localized osteolytic LCH, followed by the axial skeleton and long bones (Azouz et al., 2005[2]). In LCH affecting the long bones, the femur, humerus, and tibia are most commonly involved. Brodie's abscess, on the other hand, is a common type of subacute osteomyelitis. A characteristic feature of Brodie's abscess is the penumbra sign, seen as a rim lining of an abscess cavity with higher signal intensity than that of the main abscess on T1-weighted images (Afshar and Mohammadi, 2011[1]). However, a mass with peripheral rim enhancement and penumbra sign are usually considered features of Brodie's abscess (Bohndorf, 2001[3]). In this report, we describe a case of localized LCH of the tibia in a 2-year-old child, whose clinical manifestations and magnetic resonance imaging (MRI) findings suggested Brodie's abscess and discuss the radiologic features and differences between these two diseases. 

## Case Report

A 2-year-old girl was presented with progressive left-knee pain and swelling since 1 month. She had no history of fever, chills, or recent trauma. The patient's travel and family history were insignificant. Physical examination showed swelling, mild tenderness, and local redness in the anterior part of her left knee. Exophthalmos, skin rash, and mucosal ulcers were not observed. While the patient's white blood cell count and eosinophil count was normal, her erythrocyte sedimentation rate (ESR, 33 mm/h) and C-reactive protein (CRP, 6 mg/L) level were elevated.

**Figure 1:**
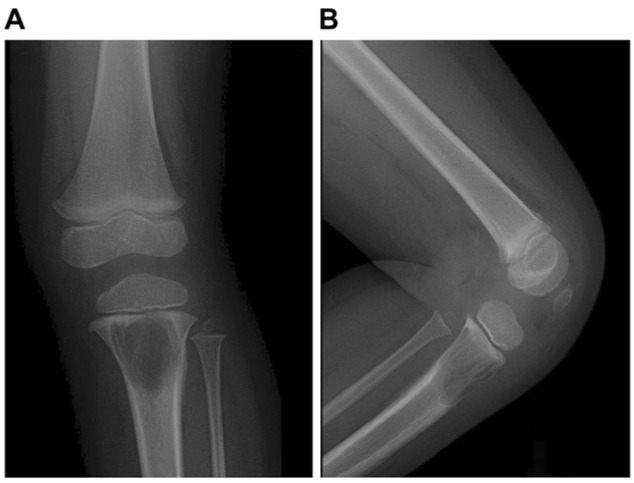
A 2-year-old girl with left knee pain and a medullary lytic lesion in the proximal tibial metaphysis. (A) Antero-posterior view, (B) Lateral view

**Figure 2:**
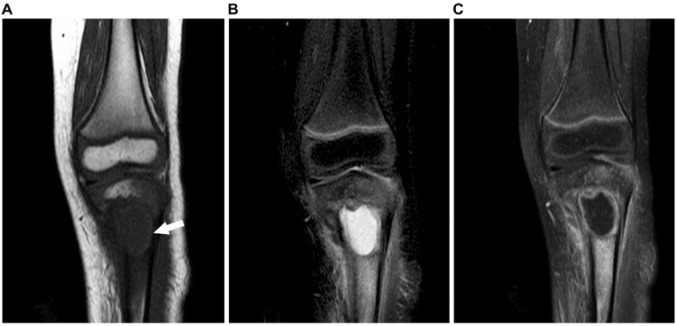
Magnetic resonance imaging findings. (A) The T1-weighted image revealed the penumbra sign (white arrow). (B) The fat-suppressed T2-weighted image revealed very high signal intensity of the central part of the lesion. (C) The gadolinium-enhanced fat-suppressed T1-weighted image showed peripheral rim enhancement.

Conventional radiography revealed a 2.5-cm-diameter medullary lytic lesion located centrally in the proximal left tibial metaphysis (Figure 1(Fig. 1)). The radiolucent process was partially ill defined, without a sclerotic margin. Cortical thinning and variation along with endosteal scalloping and mild laminated periosteal reaction were also seen. An MRI revealed a well-defined cystic lesion with a lobulated rim. This central part of the lesion was isointense compared to the surrounding muscle on non-enhanced T1-weighted images (Figure 2A(Fig. 2)), very hyperintense on fat-suppressed T2-weighted images (Figure 2B(Fig. 2)), and not enhanced after gadolinium administration (Figure 2C(Fig. 2)). The rim lining of the cavity was mildly hyperintense on T1-weighted images (Figure 2A(Fig. 2), white arrow), hypointense on fat-suppressed T2-weighted images, and enhanced after gadolinium administration. Prominent popliteal lymph nodes, extensive marrow edema, and juxtacortical deep soft-tissue patchy edema were observed surrounding the lobulated lesion margin. 

**Figure 3:**
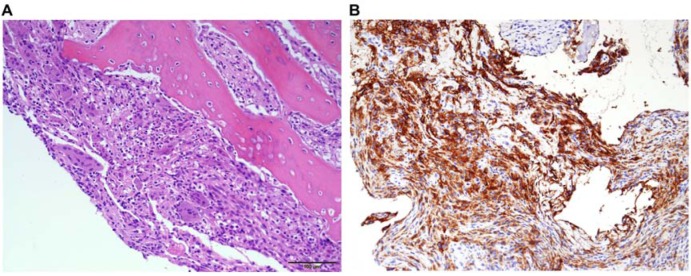
Histologic and immunohistological examination. (A) Histologic examination showed multinucleated giant cells and mononuclear histiocytes aggregated with eosinophils and neutrophils (200×). (B) Immunohistologic stains for CD1a confirmed the presence of multinucleated giant cells and mononuclear histiocytes (200×).

A tentative diagnosis of Brodie's abscess was made based on the imaging findings and clinical information; therefore, open biopsy with frozen section procedure was performed, followed by intralesional curettage and bone grafting. Intraoperative findings showed an intraosseous cystic lesion containing clear fluid. Gram staining and bacterial culture yielded negative results. Histologic examination of the specimen showed multinucleated giant cells and mononuclear histiocytes aggregated with eosinophils and neutrophils (Figure 3A(Fig. 3)). Immunohistologic labeling with antibody to CD1a and S100 protein was positive for multinucleated giant cells and mononuclear histiocytes (Figure 3B(Fig. 3)). Both histologic examination and immunohistologic labeling confirmed the diagnosis of LCH. The symptoms improved and completely resolved after surgical treatment. No evidence of recurrence was found at the 1- and 3-month radiographical follow-up examination. Post-operative whole body bone scan also showed no scintigraphic evidence to suggest the presence of polyostotic lesions.

## Discussion

Localized LCH is an uncommon disease, occurring in less than 1 % of patients with biopsied primary bone tumors (David et al., 1989[5]) and typically during the first 2 decades of life. Its peak incidence occurs between the ages of 5 and 15 years. While localized LCH can occur in region of the body, it most commonly affects the skull (approximately 50 %), pelvis, spine, mandible, and ribs (Azouz et al., 2005[2]). About one-third of all single-bone lesions involve the long bones, most commonly the femur, followed by the humerus and tibia. Long-bone involvement is more frequent in children and most lesions arise in the diaphysis (58 %), metaphysis (28 %), metadiaphysis (12 %), or epiphysis (2 %) (Stull et al., 1992[14]).

The plain radiographic appearance of localized LCH lesions depends on the phase of the disease and the site of involvement. In the acute stage, bony lesions may have an aggressive pattern of osteolysis and appear permeative with a wide zone of transition and a laminated periosteal reaction. In the chronic stage, lesions have a more benign appearance with well-defined sclerotic margins, a narrow zone of transition, and a mature or absent periosteal reaction (Kilborn et al., 2003[9]). The plain radiographic appearance of Brodie's abscess generally appears as a metaphyseal lytic lesion with a sclerotic rim but can have a markedly varied appearance as well (Dabov, 2012[4]). Plain radiographs in our case showed a metaphyseal lytic lesion with a wide medullary cavity, cortical variation, endosteal scalloping, and mild laminated periosteal reaction but no well-defined sclerotic rim. These radiographic features are suggestive of an aggressive pattern, and the differential diagnosis includes acute-stage localized LCH, osteomyelitis, leukemia, and Ewing sarcoma. Because of the clinical symptoms and signs of this patient, we could not completely exclude Brodie's abscess from the diagnosis. 

Typically, compared to radiography, MRI is superior in depicting the extent of the lesion, evaluating marrow edema, and assessing soft tissue extent; however, in this case, the MRI findings of localized osseous LCH was non-specific. The most common MR appearance of skeletal LCH is a focal lesion, surrounded by an extensive, ill-defined bone marrow and soft tissue reaction with low signal intensity on T1-weighted imaging and high signal intensity on T2-weighted imaging, considered to represent bone marrow and soft tissue edema, respectively (Moon et al., 2009[12]). While edema of adjacent bone marrow and soft tissue is particularly seen in the acute phase lesions, the chronic phase lesion is localized with decreased adjacent edema (Azouz et al., 2005[2]; Monroc et al., 1994[11]). The typical MR appearance of Brodie's abscess is a metaphyseal, well-defined intraosseous lesion with peripheral enhancement. In our case, the central part of the lesion appeared as an intermediate signal intensity on T1-weighted imaging and very high signal intensity on T2-weighted imaging as well as rim enhancement after gadolinium administration, which is typical in Brodie's abscess but uncommon for localized LCH. In addition, the lesion on unenhanced T1-weighted imaging appeared as a rim of relatively hyperintense signal intensity, which suggested the presence of a penumbra sign. The penumbra sign is not pathognomonic but a highly specific sign for Brodie's abscess (McGuinness et al., 2007[10]). While the penumbra sign has been reported in cases of localized LCH, chondrosarcoma, benign cystic lesions of the bone, and intraosseous ganglion (Davies and Grimer, 2005[6]), localized LCH accompanying the penumbra sign has rarely been validated with images. Bone marrow edema may be identified adjacent to a wide variety of intraosseous lesions including neoplastic, inflammatory, traumatic, degenerative, and ischemic conditions. The most common benign bone lesions showing adjacent edema are chondroblastoma, osteoid osteoma, osteoblastoma, and localized LCH. Malignant lesions frequently surrounded by bone marrow edema include metastasis, osteosarcoma, chondrosarcoma, and Ewing sarcoma. Benign bone lesions often show a greater amount of surrounding marrow edema than malignant lesions (James et al., 2008[8]). The diffuse surrounding marrow edema in our case may be suggestive of a benign process rather than a malignant lesion and both Brodie's abscess and acute phase localized LCH can present it. Further, the clinical symptoms and signs of this patient included progressive knee pain, local signs of inflammation, and elevated ESR levels. Therefore, we initially diagnosed Brodie's abscess in our patient.

As with many signs in imaging, the penumbra sign is not pathognomonic for Brodie's abscess and subacute osteomyelitis (Davies and Grimer, 2005[6]). Acute phase localized LCH is often confused clinically with infection, because patients may have low-grade fever, elevated ESR, mild leukocytosis, and normochromic anemia, findings seen in cases of osteomyelitis and Brodie's abscess (Stull et al., 1992[14]; Schlesinger et al., 1986[13]). Even though the clinical presentations, laboratory tests, and MRI findings (penumbra sign) may resemble those in Brodie's abscess, the differential diagnosis of acute phase localized LCH should also be considered when a cystic lesion without a sclerotic rim is found, especially among the pediatric population.

## Financial interests

None declared.

## Conflicts of interest

The authors declare that they have no conflict of interest.
